# RNA 1 and RNA 2 Genomic Segments of Chronic Bee Paralysis Virus Are Infectious and Induce Chronic Bee Paralysis Disease

**DOI:** 10.1155/2015/423493

**Published:** 2015-10-25

**Authors:** Ibrahim Youssef, Frank Schurr, Adeline Goulet, Nicolas Cougoule, Magali Ribière-Chabert, Hervé Darbon, Richard Thiéry, Eric Dubois

**Affiliations:** ^1^Anses Sophia Antipolis, Unit of Bee Pathology, 105 route des Chappes, CS 20111, 06902 Sophia Antipolis, France; ^2^Architecture et Fonction des Macromolécules Biologiques (AFMB), Aix Marseille University, CNRS, UMR 7257, Case 932, Campus de Luminy, 163 avenue de Luminy, 13288 Marseille Cedex 09, France

## Abstract

Chronic bee paralysis virus (CBPV) causes an infectious and contagious disease of adult honeybees. Its segmented genome is composed of two major positive single-stranded RNAs, RNA 1 (3,674 nt) and RNA 2 (2,305 nt). Three minor RNAs (about 1,000 nt each) have been described earlier but they were not detected by sequencing of CBPV genome. In this study, the results of *in vivo* inoculation of the two purified CBPV major RNAs are presented and demonstrate that RNA 1 and RNA 2 are infectious. Honeybees inoculated with 10^9^ RNA copies per bee developed paralysis symptoms within 6 days after inoculation. The number of CBPV RNA copies increased significantly throughout the infection. Moreover, the negative strand of CBPV RNA was detected by RT-PCR, and CBPV particles were visualized by electronic microscopy in inoculated honeybees. Taken together, these results show that CBPV RNA 1 and CBPV RNA 2 segments can induce virus replication and produce CBPV virus particles. Therefore, the three minor RNAs described in early studies are not essential for virus replication. These data are crucial for the development of a reverse genetic system for CBPV.

## 1. Introduction

Honeybee (*Apis mellifera*) is one of the most important pollinators with a big impact on agriculture and economics [1]. Honeybee is susceptible to a wide variety of environmental threats (climate, urbanization, predators, pesticides,…) and diseases. The chronic bee paralysis virus (CBPV) is responsible for a contagious and infectious disease of adult honeybee that can lead to mortalities, the chronic paralysis disease. This pathology is characterized by clusters of trembling, flightless, crawling bees, and individual black, hairless bees standing at the hive entrance [2].

CBPV can be transmitted by two main routes: (i) contact between infected and noninfected bees [3] and (ii) spread of infectious particles in the feces of paralyzed bees that are taken up orally by healthy nest mates [4]. CBPV, first isolated in 1963 [2], has anisometric particles that measure 30–60 nm in length and 20 nm in width [5]. It is a positive single-stranded RNA virus and its genome is composed of two major RNAs, RNA 1 (3,674 nucleotides [nt]) and RNA 2 (2,305 nt) [6]. The genomic RNAs are not polyadenylated at their 3′-end and have a CAP structure at the 5′-end. RNA 1 and RNA 2 encode three and four overlapping Open Reading Frames (ORFs), respectively. RNA 1-ORF3 is predicted to encode the RNA-dependent RNA polymerase (RdRp) [6]. RNA 2-ORF2 and RNA 2-ORF3 are thought to encode two structural proteins (hSP and pSP, resp.) [7]. To date, CBPV remains unclassified by the International Committee on Taxonomy of Viruses (ICTV) (http://www.ictvonline.org/). Only RNA 1-ORF3 shows similarities with Nodaviridae and Tombusviridae family [6]. Runckel et al. [8] discovered new viral sequences (Lake Sinai 1 virus and Lake Sinai 2 virus) highly related to CBPV which might belong to the same viral family. Moreover, Schuster et al. [9] described a new virus named mosinovirus related to LSV and to CBPV. The authors suggested that these viruses could belong to a new viral family.

In addition to the two major RNAs, three minor RNAs have been described in the early studies of CBPV [10]. However, these minor RNAs were not reported in any subsequent study. They were not visualized on gel and were not detected by sequencing [6]. Recently, it has been shown that total RNA obtained from purified CBPV particles is infectious after direct inoculation in honeybee [11]. However, since total RNA preparation can contain both major and minor RNAs, it was not possible to conclude about the possible role of the minor RNAs. Besides, the determination of the genetic elements essential to recover an infectious virus is needed in order to establish a reverse genetic system for CBPV.

In this study, our goal was to determine whether CBPV RNA 1 and CBPV RNA 2 inoculated together were sufficient to induce the virus replication and the chronic paralysis disease. After purification of the two major RNAs by gel electrophoresis, emerging bees were inoculated with 10^9^ RNAs copies per bee. The infectivity of CBPV major RNAs was demonstrated by observation of the clinical signs of CBPV infection, quantification of the viral genome by RT-qPCR throughout infection, detection of the antigenomic strand of CBPV RNA, and observation of CBPV viral particles by electron microscopy.

## 2. Materials and Methods

### 2.1. Maintenance of Honey Bee Colonies

This study was performed in January 2015. The queens were changed six months before the experiment and the new colonies were placed in new hives equipped with new frames. In October 2014, these hives were placed in a room heated to 32°C and were supplied by sucrose syrup complemented with protein L (Calier Laboratory, Spain). The absence of CBPV and other bee viruses was checked regularly by RT-PCR.

### 2.2. Virus Purification

Five- to seven-day-old honeybees (450 emerging bees) collected from CBPV-free colonies were used to produce CBPV by injection of the A79-P isolate (NC_010712.1). After observation of chronic paralysis signs (5 to 6 days after inoculation), CBPV particles were purified from the heads of bees by ultracentrifugation on a 10 to 40% (w/v) sucrose gradient as previously described by Olivier et al. [6].

### 2.3. CBPV Major RNAs Purification

The genomic RNAs (naked RNAs) were extracted from the purified A79-P isolate using the High Pure Viral RNA Kit (Roche) and recovered in 50 *μ*L of RNAse-free water. Extracted RNA was denatured by heating at 70°C and chilled for 5 min at 4°C and submitted to electrophoresis on a 1% low melting agarose gel using MOPS/formaldehyde buffer during 4.5 h. The RNA 1 and RNA 2 fragments were cut from the gel and extracted using QIAquick Gel Extraction Kit (Qiagen).

### 2.4. Experimental Inoculation: Sample Preparation and Collection

Emerging honeybees were collected from a CBPV-free colony. They were maintained for 5 to 7 days at 32°C in small cages providing* ad libitum *sugar candy and sucrose syrup complemented with protein L (Calier Laboratory, Spain). Eight bees from each cage were collected before the inoculation as negative controls. The remaining bees were anaesthetized with carbon dioxide (CO_2_) and inoculated with 4 *μ*L of phosphate buffer (PB, pH 7) suspension containing whole CBPV particles (A79-P isolate), naked RNA, or major RNA mixture* via* intrathoracic injection (Table 1). Immediately after inoculation, 8 bees from each experimental condition were sampled to quantify the RNA loads. The bees were then incubated at 32°C for 6 days and the clinical signs were observed and recorded daily. Asymptomatic, symptomatic, and dead bees were recovered daily and stored separately at −80°C.

### 2.5. RNA Extraction and cDNA Synthesis

Bees were individually crushed in 1 mL of 0.01 mM PB. After two clarifications of the homogenate by centrifugation (10 min at 8,000 ×g), the viral RNA was extracted using the High Pure Viral RNA Kit (Roche) according to the manufacturer's recommendations. The quantity and quality of purified RNA were estimated by measuring the optical density at 260 nm and 280 nm. Complementary DNA was synthesized by reverse transcription as described by Ribière et al. [12].

### 2.6. CBPV Major RNA Quantification by Real-Time Reverse-Transcription PCR (RT-qPCR)

The RNA 1 fragment was quantified by RT-qPCR as described by Blanchard et al. [13]. A new RT-qPCR was set up for RNA 2 quantification. CBPV RNA 2 sequences available in GenBank were aligned, and primers and probes were designed using the Primer Express software (Applied Biosystems) in the conserved region. Because no consensus sequences were found, several sequences were selected for each primer and the probe. The selected primers amplify a fragment (72 to 74 nt long) and are located in the coding region of the predicted structural protein pSP (RNA 2, ORF3). The forward, reverse primers and TaqMan probes are detailed in Table 2. The probes were labeled with the fluorescent reporter dye FAM (6-carboxyfluorescein) at the 5′-end and with the fluorescent quencher dye TAMRA at the 3′-end.

The number of RNA copies was estimated using 5 *μ*L cDNA by RT-qPCR carried out on a 7500 Real-Time PCR system (Applied Biosystems, USA). Briefly, the PCR reaction was performed in duplicate in a MicroAmp Optical 96-Well Plate, containing 1X TaqMan Universal PCR Master Mix with uracil-N-glycosylase (UNG) (Applied Biosystems, USA), 320 nM of each primer mix (forward and reverse primer mix), 200 nM of the qCBPV probe mix, 1X Exo Internal Positive Control (IPC) Mix VIC (Applied Biosystems, USA), 1X Exo IPC DNA (Applied Biosystems, USA), and 5 *μ*L of standard template (10^8^ to 10^2^ DNA copies of pGEM-T Easy [Promega] recombinant plasmid containing the pSP sequence) or cDNAs in a total volume of 25 *μ*L. The thermal cycling conditions were 2 min at 50°C (active temperature for UNG to degrade any carryover DNA amplified from previous reactions) and 10 min at 95°C (activation of AmpliTaq Gold DNA Polymerase and degradation of UNG), followed by 40 cycles of denaturation at 95°C for 15 s and annealing/extension at 60°C for 1 min.

### 2.7. Detection of Bee Viruses

Before the inoculation, a multiplex RT-PCR [14] was performed to verify the absence of other bee viruses using previously published primers: black queen cell virus (BQCV) [15], acute bee paralysis virus (ABPV) [16], deforming wing virus (DWV) [17], Israeli acute bee paralysis virus (IAPV) [18], and sacbrood virus (SBV) [19]. In addition, bees were tested for Lake Sinai virus by RT-PCR according to Runckel et al. [8].

### 2.8. Detection of CBPV Negative RNA

To assess the CBPV replication, we used a specific RT-PCR to detect the negative strand form of CBPV RNA as developed by Celle et al. [20].

### 2.9. Transmission Electron Microscopy

The formation of viral particles was checked using electron microscopy. All RNA samples of bees inoculated with CBPV major RNAs showing a load of >10^10^ RNAs copies per *μ*L were mixed together. After addition of 2.5 mL carbon tetrachloride (CCl4), homogenates were incubated for 1 h at 4°C and then clarified at 200 ×g for 10 min at 4°C. Aqueous phases were recovered and centrifuged at 1,700 ×g for 1 h at 4°C. Ultracentrifugation of supernatants was carried out at 75,000 ×g for 3.5 h at 4°C. The pellet was resuspended in 50 *μ*L PB. All the samples were diluted with water to 0.1 mg/mL, the negative control was diluted with the same 1/25 ratio, and 3.5 *μ*L drops were applied onto glow-discharged formvar-carbon coated grids (Agar Scientific). After 1 min incubation at room temperature, the liquid excess was blotted off and the grids were rinsed with water before being stained with uranyl acetate 1%. Grids were transferred into a FEI Tecnai Spirit G2 Electron Microscope operated at 120 kV and imaged with an EAGLE 2kX2k CCD camera at a nominal magnification of 18,000x and with an underfocus of approximately 2.5 *μ*m.

## 3. Results

### 3.1. CBPV Major RNA Purification

**Figure 1:**
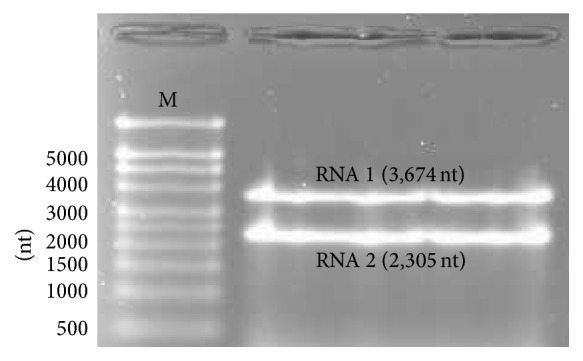
Gel electrophoresis separation of RNA 1 and RNA 2 segments of CBPV genome (A79-P isolate). 1X MOPS/formaldehyde 1% agarose gel. M: millennium RNA marker (Ambion).

The total RNA extracted from the purified CBPV particles (A79-P isolate) was run on a MOPS/formaldehyde gel to separate RNA 1 and RNA 2 (Figure 1). After the electrophoresis, only major RNAs were visualized. No fragment of about 1,100 base long was visualized. The fragments of RNA 1 and RNA 2 were cut from the gel and purified, and their amounts were calculated by RT-qPCR. The quantity estimated of purified RNA 1 was about 2.1 × 10^10^ copies per *μ*L and purified RNA 2 was about 8.3 × 10^10^ copies per *μ*L. Each RNA was diluted to 1.0 × 10^9^ RNAs copies per *μ*L. Then, RNA 1 and RNA 2 were mixed together (major CBPV RNAs) to be inoculated to the bees.

### 3.2. Validation of the CBPV RNA 2 RT-qPCR

**Figure 2:**
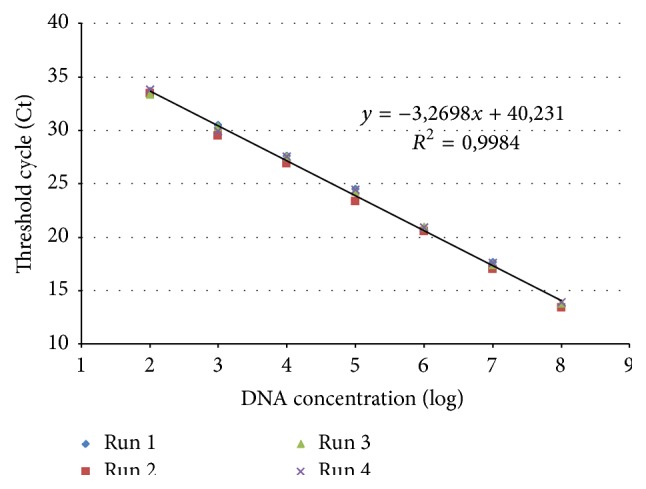
DNA standard curve of CBPV RNA 2 RT-qPCR obtained with a 10-fold serial dilution (10^8^ to 10^2^ DNA copies per reaction) of 2,710 kb plasmid including the coding sequence of the predicted structural protein pSP on RNA 2-ORF3. Four independent runs were performed and allowed to obtain the linear regression analysis of the Ct measured for each amplification (*y*-axis) versus log_10_ of DNA concentration of each dilution (*x*-axis). The equation of the linear regression and the correlation coefficient (*R* ^2^) are indicated.

In order to evaluate the CBPV RNA 2 RT-qPCR, four independent runs were performed using a 10-fold serial dilution of a plasmid DNA control as standard. The standard curve (Figure 2) showed a linear correlation between Ct and log_10_ DNA concentration of each run (*R* ^2^ = 0.998). The slope of the DNA standard curve was −3.27 and the average efficiency of RNA 2 RT-qPCR was 102%. The limit of quantification was 100 DNA copies per reaction.

### 3.3. Symptoms of Chronic Bee Paralysis

**Figure 3:**
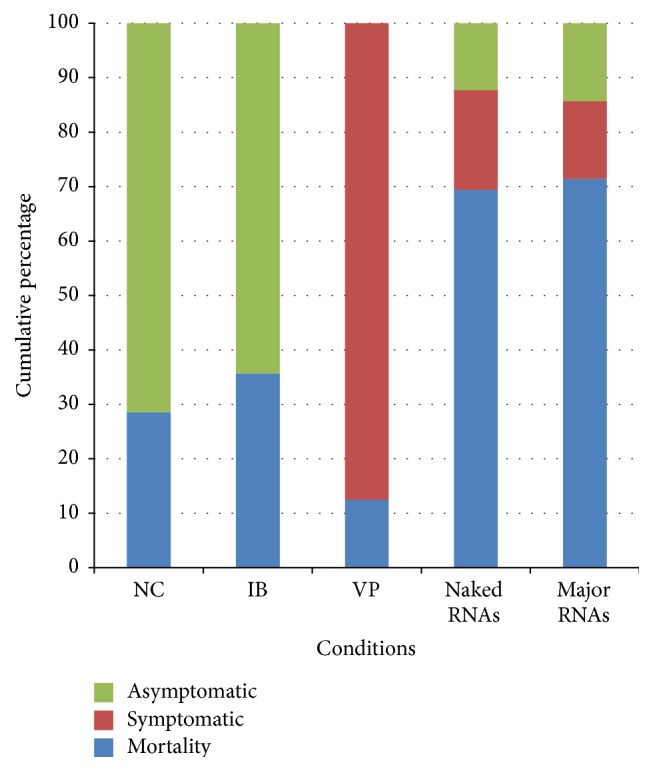
Cumulative percentage of dead, symptomatic, and asymptomatic bees after different treatments 6 days after inoculation: Negative control (NC), CBPV-free inoculation buffer (IB), positive control: CBPV particles (VP), CBPV naked RNAs, and CBPV major RNAs.

Bees were exposed to five different treatment groups including (i) bees not inoculated, (ii) bees inoculated with PB, (iii) bees inoculated with CBPV particles, (iv) bees inoculated with CBPV naked RNAs, and (v) bees inoculated with CBPV major RNAs (Table 1). CBPV clinical signs were observed and dead bees were collected daily.  Figure 3 shows the cumulative percentages of dead, symptomatic, or asymptomatic bees observed in each experimental condition after 6 days of inoculation. Bees not inoculated (NC) and those inoculated with PB buffer only (inoculation buffer: IB) did not show any signs of chronic paralysis throughout the experiment. However, 28% of bees not inoculated (4 bees out of 14) and 35% inoculated with the buffer (5 bees out of 14) were dead during the first days of the assay (2 and 4 days post-inoculation [dpi]). The bees inoculated with CBPV particles (VP) showed chronic paralysis signs (trembling, crawling) within 5 and 6 dpi. About 12.5% of bees were dead at 2 and 4 dpi and 87.5% were symptomatic. In the group of bees inoculated with naked RNAs, 69% of bees were dead 6 dpi, 19% of bees showed chronic paralysis clinical signs, and 12% bees were asymptomatic. In the group of bees inoculated with CBPV major RNAs, 71.4% of bees were dead within 6 dpi, 14.2% of bees showed the chronic paralysis clinical signs, and 14.2% bees were asymptomatic. Based on a chi-squared test, a significant difference in the number of symptomatic and dead bees was found between control and inoculated bees (*p* < 0.001). However, no significant difference in symptoms and mortality was found between bees inoculated with CBPV naked RNAs and those inoculated with CBPV major RNA (*p* = 0.766). This result confirms that the clinical signs observed could be associated with RNA inoculations (naked RNA as well as major RNA).

In addition, two other treatments were tested: bees inoculated with CBPV RNA 1 or with CBPV RNA 2. Around 12.5% (5 bees out of 40 bees) of bees inoculated with CBPV RNA 1 and 25% (7 bees out of 40 bees) of those inoculated with CBPV RNA 2 were dead. However, no chronic bee paralysis symptoms were observed (data not shown).

### 3.4. Quantification of CBPV Major RNAs in Inoculated Bees

**Figure 4:**
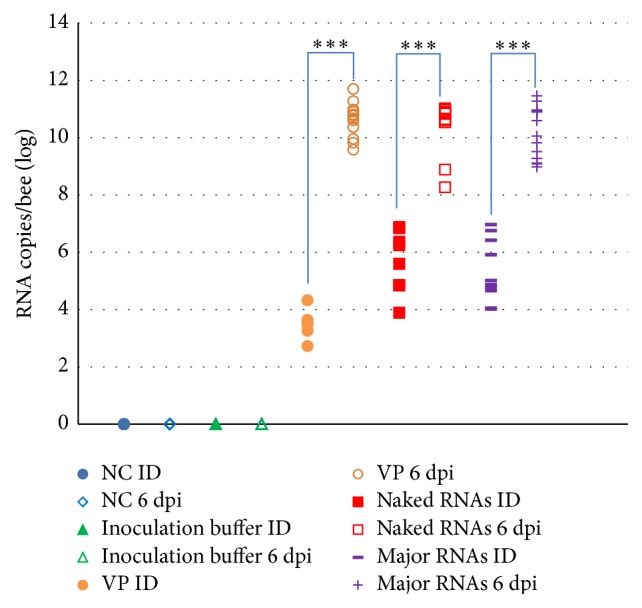
CBPV RNA 1 quantification in individual inoculated bees. NC: negative control, VP: bees inoculated with CBPV viral particles, CBPV naked RNAs, and CBPV major RNAs. This graph shows the results of the RT-qPCR of CBPV RNA 1. Significant differences between inoculation day (ID) and 6 days post-inoculation (dpi) for each condition are indicated (*∗∗∗*) (*p* value < 0.001, Mann-Whitney test).

The number of copies of RNA 1 and RNA 2 was estimated using RT-qPCR methods.  Figure 4 shows the RNA 1 copies number per bee after different treatments at inoculation day (ID) and 6 days post-inoculation (6 dpi). The negative control (NC) and IB-treated bees remained negative for CBPV throughout the infection, whereas the RNA copy number increased significantly when bees were inoculated with VP, naked RNAs, and major RNAs between ID and 6 dpi. At 6 dpi, the symptomatic and dead bees inoculated with CBPV major RNAs showed a similar CBPV RNA load as those inoculated with CBPV particles and CBPV naked RNAs (10^10^–10^11^ RNA 1 copies/bee). In contrast, the asymptomatic bees inoculated with naked or major CBPV RNAs presented a lower amount, ranging from 10^7^ to 10^8^ RNA copies/bee. Bees inoculated with CBPV RNA 1 or CBPV RNA 2 only did not show any increase of the viral load throughout the infection (data not shown).

**Figure 5:**
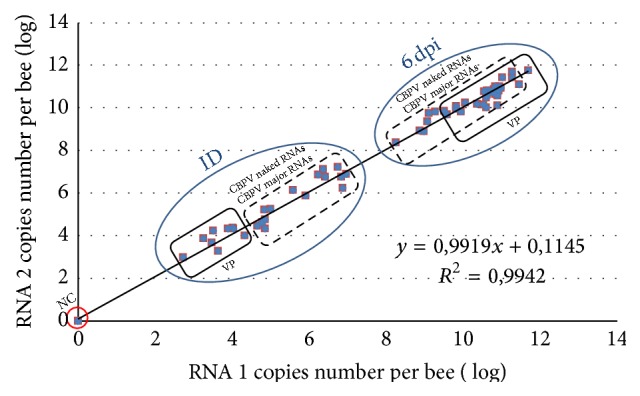
Linear regression analysis of the results from RT-qPCR quantification of RNA 1 versus RNA2. The equation of the curve and the correlation coefficient are indicated. The results at inoculation day (ID) and those at 6 days post-inoculation (6 dpi) are represented in a blue circle. The RNA copies number of bees inoculated with CBPV viral particles (VP) is represented by a rectangle, bees inoculated with naked RNAs and major RNAs by dotted rectangle, and bees not inoculated as negative control (NC) by a red circle.

The quantities of RNA 1 and RNA 2 copies per bee were estimated using the two RT-qPCR methods (Figure 5). The results showed that there is no significant difference between RNA 1 and RNA 2 copy numbers in the bees. The slope of the regression curve is 0.9919 and the intercept is 0.11.

### 3.5. CBPV Replicative Strand Detection by Strand-Specific RT-PCR

**Figure 6:**
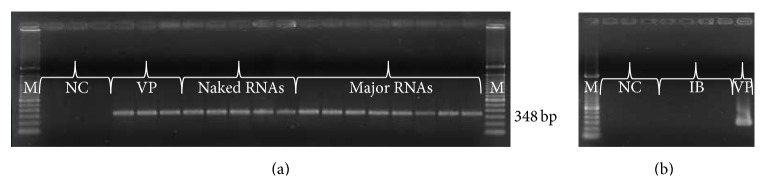
Antigenomic RNA detection by specific RT-PCR. 1.2% agarose gel electrophoresis of the PCR products (amplicons). (a) Three samples of negative control (NC) and three samples of bees inoculated with CBPV particles (VP), five samples of bees inoculated with CBPV naked RNAs, and eight samples of bees inoculated with CBPV major RNAs. (b) Three samples of negative control (NC), four samples of bees inoculated with inoculation buffer (IB), and one sample of bees inoculated with viral particle (VP) were tested. M: 100 bp DNA marker (TrackIt, Invitrogen).

The replicative RNA strand was tested by a specific RT-PCR on several samples from each condition studied (Figure 6). Interestingly, the CBPV antigenomic strand was detected in the bees inoculated with CBPV particles (VP), naked RNAs, and major RNAs (Figure 6(a)), whereas it was not detected neither in bees not inoculated (NC) nor in bees inoculated with the phosphate buffer (IB) (Figure 6(b)).

### 3.6. Assembly of CBPV Particles

**Figure 7:**
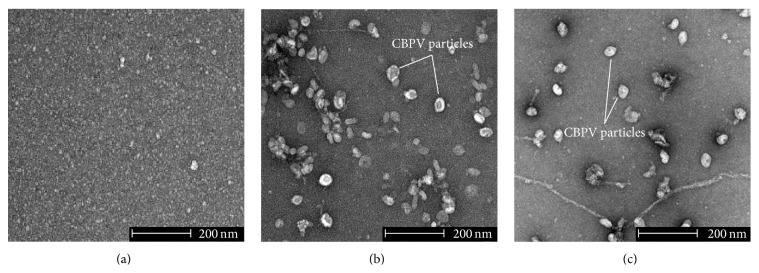
Transmission electron microscopy of homogenates from experimented bees. (a) Negative control bees, (b) positive control bees (inoculated with CBPV particles), and (c) bees inoculated with CBPV major RNAs.

Transmission electron microscopy was used to confirm the formation of viral particles (Figure 7). Six days after inoculation, bees inoculated with CBPV major RNAs show the presence of anisometric viral particles (Figure 7(c)). These particles, around 50 nm in length, are physically identical to CBPV purified particles, the positive control (Figure 7(b)). Moreover, to ensure that the formation of the viral particles is due to the inoculation with CBPV major RNAs, not inoculated bees (negative control) were examined; no CBPV particles were found (Figure 7(a)).

### 3.7. Absence of Other Bee Viruses

No other bee viruses were detected by conventional RT-PCR in the bees used for the experiments.

## 4. Discussion

This study demonstrates that the RNA 1 and RNA 2 segments of the CBPV genome are the essential genetic elements for CBPV replication and that they are sufficient to induce the chronic bee paralysis disease.

CBPV, a worldwide virus, infects the honeybees. The anisometric morphology of its particles is unique among bee viruses as most of them are picorna-like viruses with icosahedral particles. CBPV genome consists of positive single-stranded RNA segments. Overton et al. [10] reported that it is composed of five RNA fragments: the two major RNAs (RNA 1 and RNA 2) and three minor RNA segments: RNA 3a, RNA 3b, and RNA 3c, each of 1,100 nucleotides long. In this study, we did not detect the minor RNAs by gel electrophoresis during the purification of CBPV genome (Figure 1). This result confirms the previous conclusions from Olivier et al. [6] who sequenced the CBPV genome and did not detect any minor RNAs. These authors showed that CBPV RNA 1 and CBPV RNA 2 encode three and four overlapping ORFs, respectively. The RNA polymerase synthesis is supposed to be the result of a frame-shift mechanism between the ORF1 and ORF3 of RNA 1. Very recently, Chevin et al. [7] reported that RNA 2 might support the coding sequences of structural proteins. Kuchibhatla et al. [21] could identify homologs of most of CBPV protein thought to be orphans. Indeed, they found that ORF 1 of CBPV RNA 1 is homologous to the* alphavirus* methyltransferase guanylyltransferase. In addition, they found that ORF3 of CBPV RNA 2 shares significant similarities with a virion membrane protein found in various insect and plant viruses, and they suggested that ORF2 of CBPV RNA 2 may be a virion glycoprotein.

Recently, Chevin et al. [11] showed that CBPV total RNAs purified from infected bees induce the replication of CBPV in the honeybees and the formation of viral particles. However, the authors did not conclude about the genetic elements essential to the replication of CBPV. Here, we investigated further this point. Emerging honeybees were inoculated with purified CBPV major RNAs at 10^9^ RNAs copies per *μ*L. We also inoculated bees with a lower amount of CBPV major RNAs in an independent experiment (10^4^ major RNAs copies per bee) and found a significant increase of CBPV RNA level and CBPV replication in 16% of those bees (data not shown). However, no clinical signs were observed in this last experiment. Therefore, the efficiency of the infectivity depends on the amount of inoculated major RNAs.

In the current study, five different treatments were tested (Table 1). In each one, the bees were divided into three categories: symptomatic, asymptomatic, and dead bees. The bees not inoculated (NC) and those inoculated with the buffer showed, respectively, 28% and 35% of mortality throughout the infection, which represents, respectively, 4 and 5 bees out of 14 (Figure 3). In these control groups, neither CBPV (Figure 4) nor other bee viruses were found. Thus, this mortality can be due to the experimental and the artificial conditions that these bees could not stand.

Bees inoculated with major RNAs showed the signs of chronic paralysis disease within 6 days after inoculation and 87.5% of the bees were dead or symptomatic (Figure 3), suggesting that CBPV major RNAs are infectious. Moreover, CBPV genomic load increased significantly after 6 days of inoculation with CBPV major RNAs (Figures 4 and 5), up to approximately 10^12^ RNAs copies/bee in dead bees. No other bee virus could be detected by conventional RT-PCR indicating that the mortality and the clinical signs were associated with CBPV replication. The amount of CBPV RNAs needed to induce chronic paralysis is much higher when using major RNAs (4.0 × 10^9^ CBPV copies per bee) compared to that needed when using the CBPV particles (4.0 × 10^4^ CBPV copies per bee). This may be due to several reasons: a lower stability of purified RNAs compared to native RNA protected by the viral capsid, partial degradation of RNAs during the purification process, and/or lower efficiency of infection through RNA transfection compared to that obtained with the virus. The infection by the native virus particles probably occurs* via* interaction with a cell receptor, which may facilitate entry into cells.

CBPV is a positive single-stranded RNA virus, so the synthesis of antigenomic RNA (negative strand) is carried out during viral replication [19]. The negative strand form was detected in bees inoculated with CBPV particles, naked RNAs, and major RNAs. In addition, anisometric particles similar to CBPV particles were observed by electron microscopy after the infection of bees with CBPV major RNAs. These results along with those of RT-qPCR demonstrate that CBPV major RNAs could induce CBPV genome replication and virus particles formation in the honeybees. In conclusion, this study shows that CBPV RNA 1 and CBPV RNA 2 are infectious and sufficient to induce genomic replication, virus production, and the chronic paralysis disease in honeybee. Therefore, the three minor RNAs described in early studies are not essential for CBPV infection and are not part of the CBPV genome. Whether minor RNAs were subgenomic RNAs remains to be determined. It should be noticed that they were associated with the small virus-like particles associated with chronic bee paralysis virus [22] and thus may result from virus contamination by a satellite virus. Altogether, these results are crucial to develop a reverse genetic system in order to study the CBPV genome.

## Figures and Tables

**Table 1:** Experimental conditions and bee sampling.

Conditions	CBPV RNA copy number per bee	Number of bees per cage
Bees not inoculated (negative control: NC)	0	30
Bees inoculated with inoculation buffer (IB)	0	30
Bees inoculated with CBPV particles (VP)	4.0 × 10^4^	40
Bees inoculated with CBPV (naked RNAs)	4.0 × 10^9^	65
Bees inoculated with the mix of CBPV RNA 1 + RNA 2 (major RNAs)	4.0 × 10^9^	65

**Table 2:** Sequences of the primers and probes used for the CBPV RNA 2 RT-qPCR. Oligonucleotide sequences are located on RNA 2 complete genome of the A-79P isolate (NC_010712.1).

Equimolar mixture	Sequences	Position on CBPV-RNA 2 (based on A79-P isolate)
Forward primer mix	AGGCGCCGTAGCTGTTTCT	590–608 nt^1^
	GCGCCGTGGCTGTTTCT	592–608 nt

Reverse primer mix	CCCCGATCATATAAGCAAACTTCTC	637–663 nt
	CCCGATCATATAGGCAAACTTCTC	639–662 nt
	CCCCGATCATATATGCAAACTTCTC	639–663 nt

Probe mix	CTGCGGTACTCAGCTCAGCTCGACG	610–634 nt
	TGCGGTACTCAGCTCAGCTCGGC	611–631 nt

^1^nt: nucleotide.
